# Signs & Symptoms of Dextromethorphan Exposure from YouTube

**DOI:** 10.1371/journal.pone.0082452

**Published:** 2014-02-12

**Authors:** Michael Chary, Emily H. Park, Andrew McKenzie, Julia Sun, Alex F. Manini, Nicholas Genes

**Affiliations:** 1 Ichan School of Medicine at Mount Sinai, New York, New York, United States of America; 2 Rutgers New Jersey Medical School, Newark, New Jersey, United States of America; 3 Division of Medical Toxicology, Icahn School of Medicine at Mount Sinai, New York, New York, United States of America; 4 Department of Emergency Medicine, Icahn School of Medicine at Mount Sinai, New York, New York, United States of America; University of Namur, Belgium

## Abstract

Detailed data on the recreational use of drugs are difficult to obtain through traditional means, especially for substances like Dextromethorphan (DXM) which are available over-the-counter for medicinal purposes. In this study, we show that information provided by commenters on YouTube is useful for uncovering the toxicologic effects of DXM. Using methods of computational linguistics, we were able to recreate many of the clinically described signs and symptoms of DXM ingestion at various doses, using information extracted from YouTube comments. Our study shows how social networks can enhance our understanding of recreational drug effects.

## Introduction

### Motivation

This study investigated whether YouTube is a useful source of information on the recreational use of an over the counter substance whose usage is, otherwise, challenging to track. If data from social media about recreational drug use concord with clinically documented symptoms and doses, then those data could be used to explore aspects of recreational drug use, such as short-term trends, that are mostly inaccessible with current means: case reports from emergency rooms and poison center calls. This study represents an application of computational linguistics to social media to provide a new data source for healthcare professionals.

### Dextromethorphan (DXM)

Dextromethorphan (DXM) is marketed as a cough suppressant and is found in many over-the-counter (OTC) cough and cold preparations. At low dosages, it binds to the 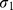 opioid receptor, which accounts for its suppression of the cough reflex [1]. At higher dosages it is metabolized to dextrorphan, an N-methyl-D-aspartate (NMDA) antagonist [2] that can produce dissociative hallucinations [3], [4], similar to phencyclidine and ketamine. In addition to dissociative effects,, tachycardia, hypertension, agitation, ataxia, and psychosis have also been reported at those higher dosages [5]–[7].

### Public Health Impact of Recreational Use of DXM

Recreational use of DXM is increasingly common. Calls to poison control centers concerning exposures to DXM sharply increased in 2006 and have remained elevated since then [8], [9]. Recreational use is prevalent among youths and young adults in the US with approximately 1 million people aged 12–25 using DXM recreationally each year [10]. This recreational use leads to approximately 6000 emergency department visits each year (approximately half of all visits are due to recreational use from those aged 12–25 [11]).

### Barriers to Collecting Data on the recreational Use of DXM

Information about the patterns of drug use traditionally comes from surveys and reports from emergency medicine physicians or poison control centers. For example, information around cocaine abuse is collected by in-person interviews done by the Substance Abuse and Mental Health Service Administration every 5 years and by a mail-in survey every year [10]. In contrast, substances such as DXM are often not as thoroughly investigated because they have recognized uses in medicine and are not perceived to be “drugs of abuse” [12]. Consequently, data on the recreational use of DXM are not collected as systematically as those on the usage patterns of illicit drugs. This makes it difficult to ascertain the public health impact of the recreational use of DXM [13]. Consequently, the ability to analyze and understand substance abuse using structured data collected by physicians and poison control centers is limited.

Nevertheless, the available data describe a picture of increasing use of DXM at doses associated with dangerous side effects. From 2005 to 2009, the sale of DXM-containing products increased by approximately 19% from 145 million bottles to 173 million bottles [14]. However, as discussed above and unlike restricted of illicit substances, DXM has recognized uses in medicine that confound the interpretation of this number. Data specific to the recreational and recreational use of DXM comes from reports of overdoses from emergency departments and exposures called to regional poison control centers. Because these reports likely involve more adverse or severe presentations, they likely do not represent the full spectrum of recreational use of DXM [12].

### Social Media as a Source of Medical Information

Social media can provide a wider spectrum of information on recreational usage of DXM. Moreover, data collection from social media is cheap and can be done in real-time or close to it. Although often thought to be limited to perfunctory discussion, social media can be used to investigate medical topics in depth. [15], [16]. For example, an analysis of the effects of *Salvia divinorum* as seen in user-generated YouTube videos first highlighted aspects of recreational drug use that are difficult to obtain by poison center calls, such as the typical amount ingested that does not result in adverse effect [17].

### YouTube

**Figure 1:**
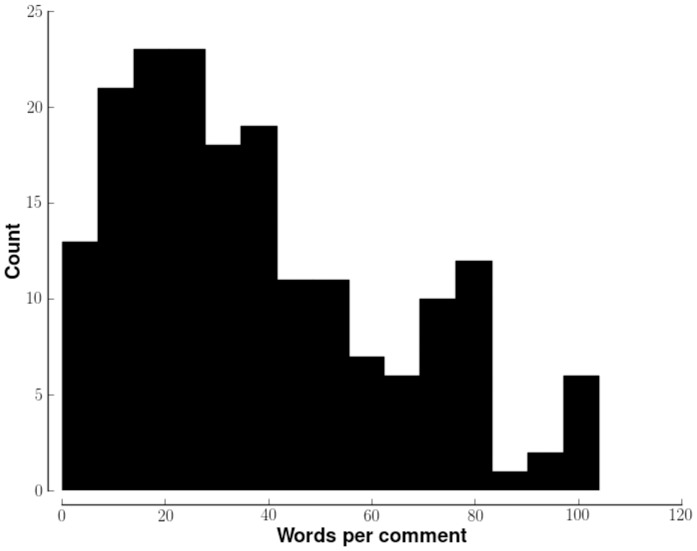
Number of words in YouTube comments. The histogram (bin size = 9 words) shows the distribution of number of words for all YouTube comments analyzed in this paper. Words were defined as strings of characters flanked by white spaces after the preprocessing described in results. The bin size for the histogram is 9 words.

YouTube is a popular social network that lets users share, arrange and comment on videos, via website or mobile apps. It receives more than 800 million unique visitors per month [18]. Unlike social media sources such as Facebook, most users who post on YouTube do not use their real names when posting. This may limit self-censorship when discussing illicit topics. Data from YouTube have been used to better understand the effects of Salvia divinorum, and the dynamics of drug education [17], [19], [20]. The corpus derived from YouTube comments has characteristics similar to other English language corpora and so, presumably, is amenable to similar analyses. Most YouTube comments have 15–20 words (Figure 1), which is comparable in length to English sentences [21].

### Term frequency-inverse document frequency (tf-idf)

**Figure 2:**
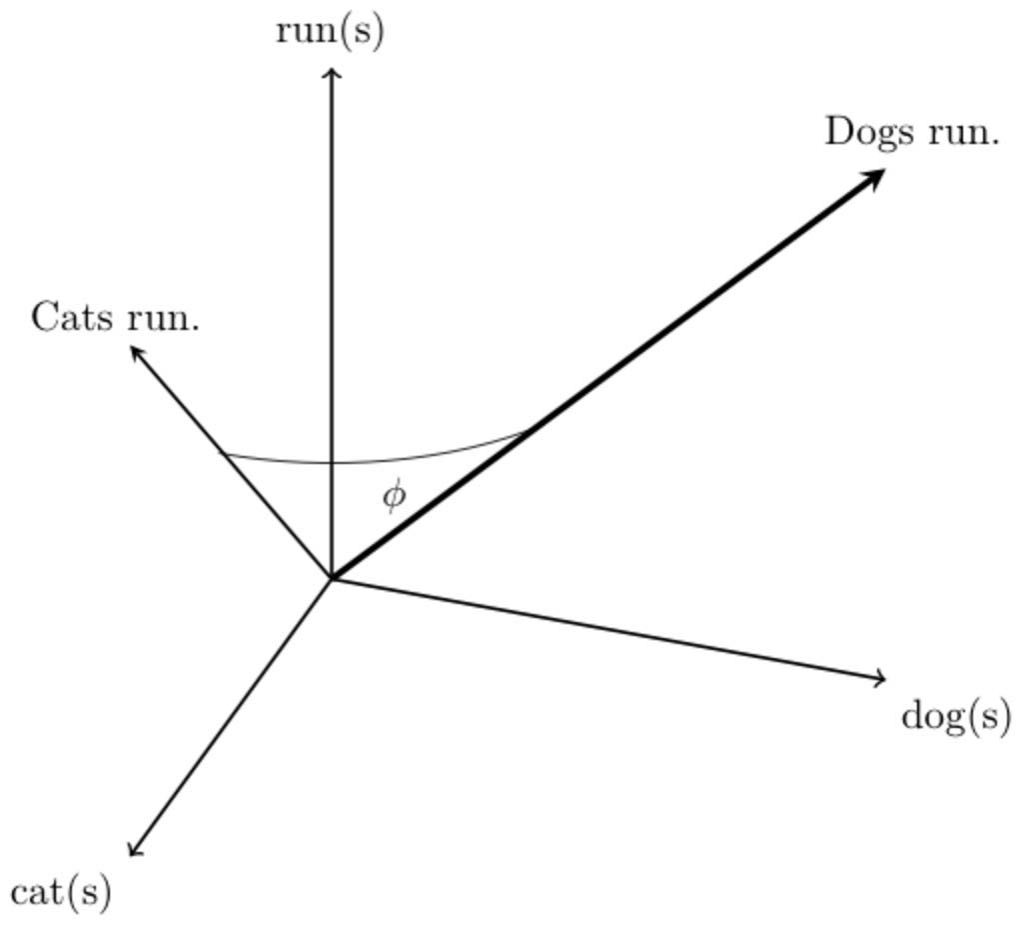
Representing sentences as vectors. The left panel shows the representation of two sentences, “Dogs run and dogs run.” and “Cats run.” as vectors. The “Dogs run” vector is twice the length of “Cats run” because each term in the former is repeated. Equation 1 illustrates how the cosine of the angle between the two vectors, 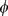, quantifies the similarity between the two sentences.

To quantitatively analyze textual data from social media, those data need to be transformed from letters to series of numbers. One common approach for converting a piece of text, termed a *document*, into numeric data is to transform the document into a vector by allowing each word to be a dimension [22]. For example, the sentence “Cats run.” becomes a three dimensional vector (Figure 2, left panel). Representing texts as vectors reduces quantifying the similarity between documents to computing the angle between vectors (Figure 2, right panel).

Representing each word as a dimension assumes that the occurrence of one word does not depend on the occurrence of any other words; this means that a document is an unordered collection, or “bag”, of words. If the order of words is important in a document, their corresponding dimensions are not orthogonal. One solution, in that case, is to use not words but combinations of words as axes. Dimensional reduction approaches, such as singular value decomposition, can be used to determine these composite axes.
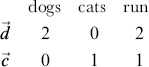


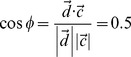
(1)


The major flaw with this approach is that it assumes that words that occur together frequently are semantically related. However, grammar and syntax often require the juxtaposition of semantically unrelated words. Removing stopwords, words that occur nonspecifically across many texts, makes word frequency a more specific indicator of semantic content, albeit still imperfect.

There are elaborations on tf-idf that attempt to disentangle semantics from word frequency [23]. These approaches create measurements of semantic similarity that are specific to each data set. Our approach is more general because it uses a widely accepted measure of semantic similarity.

### Word Net

**Figure 3:**
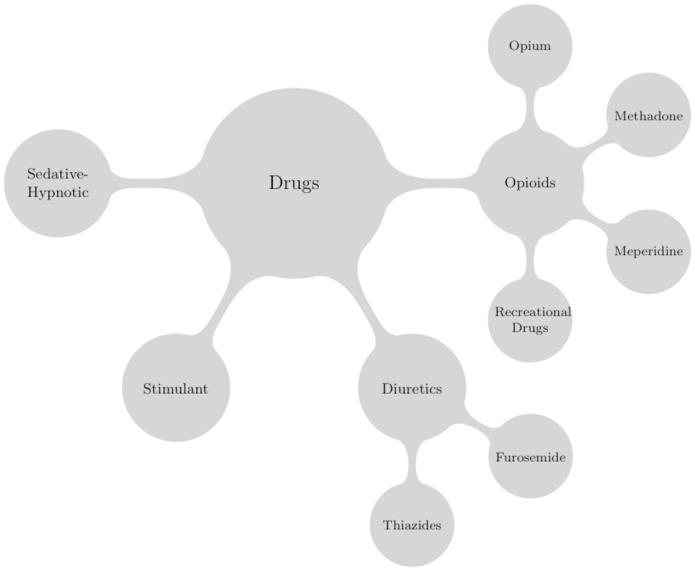
Example WordNet hierarchy. Spreading out from the root concept of drugs are progressive refinements or *hyponyms*. Counting each path as 1 and starting from the root node, Drugs, one may calculate the path similarity of any two concepts (see text).

WordNet is a graph representation of the English language that groups words with similar meanings together into clusters, which roughly correspond to concepts [24]. Figure 3 shows part of the WordNet cluster for the concept *drugs*. We use the distance between clusters in WordNet to quantify how similar two concepts are. We calculate the similarity between two words as the complement of the ratio of the shortest path length between the two nodes representing those concepts to the diameter of the graph in Figure 3. For example, meperidine's similarity to methadone is 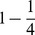 but meperidine's similarity to furosemide is 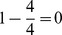. This quantification agrees with our intuition because methadone and meperidine are analgesics whereas furosemide is a diuretic.

## Results

### Overall word frequency

**Figure 4:**
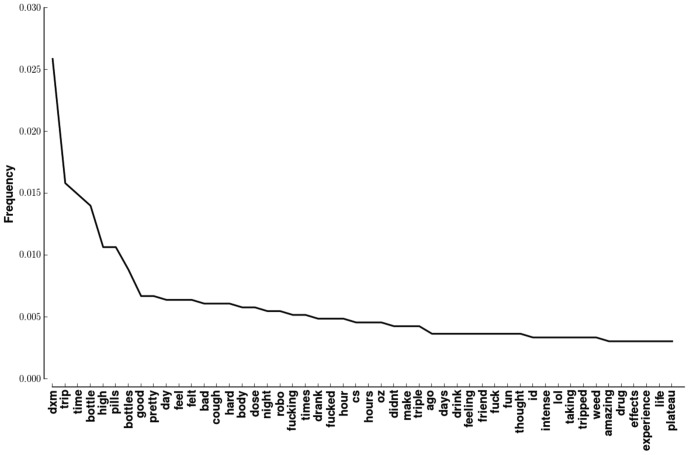
Most frequent words in YouTube comments. Probability density function of words from all YouTube comments analyzed in this paper. The frequency of occurrence was calculated after removing stopwords.

Figure 4 shows the 40 most frequent words in the corpus. The most frequent word is DXM. *Robo*, also commonly mentioned, refers to *Robitussin*, an over-the-counter cough syrup that is a source for DXM. *Cs* is a shortened form of *Triple C's*, which refers to *Coricidin Cough and Cold*. The drug with the trade name *Coricidin*, in contrast to Robitussin, contains both DXM and chlorpheniramine, an antihistamine. Some versions also contain acetaminophen and the expectorant guaifenesin. Because punctuation is removed prior to tabulation, *Id* refers to *I'd*.

### Range of dosages

**Figure 5:**
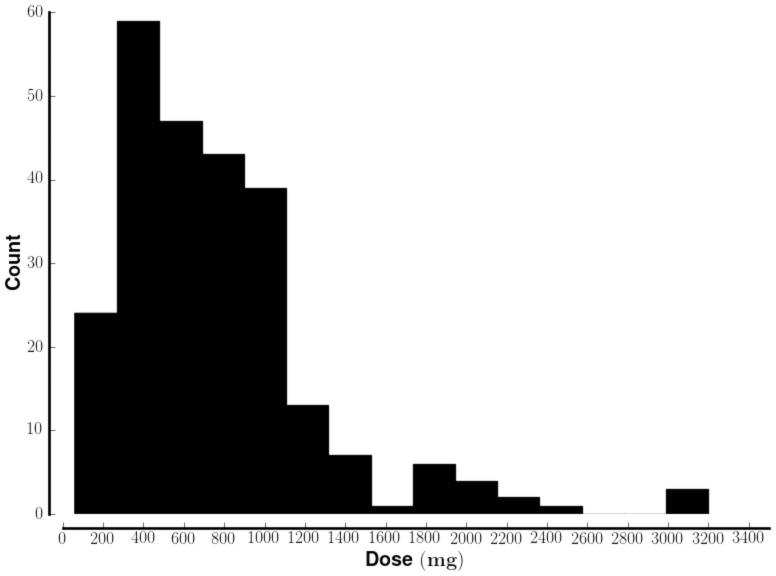
Range of dxm dosages discussed on YouTube. The histogram (bin size = 200mg) shows the distribution of dosages mentioned in the YouTube comments. All doses were converted to milligrams.

The data from YouTube (Figure 5) correspond with prior reports that DXM is most commonly ingested in amounts that range from 0 to 1500mg [25]. Distinct symptoms occur with certain ranges of dosages, termed plateaus (see [7] and [25], summarized in Table 1). The mode of the distribution in Figure 4 occurs at 375mg. This falls within the most common recreational dosage range of 200–400mg (plateau 2 in Table 1). Plateau 2 features words suggestive of alcohol and marijuana use, corresponding with the clinical description of the effects of DXM at those doses. The dropoff in dosages after 1600mg corresponds with reports of adverse affects above 1500mg. Death is associated with dosages above 1800 mg [25].

**Table 1:** Signs and symptoms associated with dextromethorphan ingestions.

Dosage Range (mg)	Signs and Symptoms
100–250 (Plateau 1)	mild stimulant effect similar to that of methylenedioxyamphetamine
250–400 (Plateau 2)	combination of concurrent ethanol and marijuana use, some experience hallucinations
450–800 (Plateau 3)	dissociative, “out-of- body” state like that produced by a low recreational dose of ketamine
>800 (Plateau (4)	fully dissociative condition similar to that produced by ketamine intoxication, death at dosages above 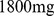

Adapted from [25].

### Semantic similarity of YouTube comments to case reports

**Figure 6:**
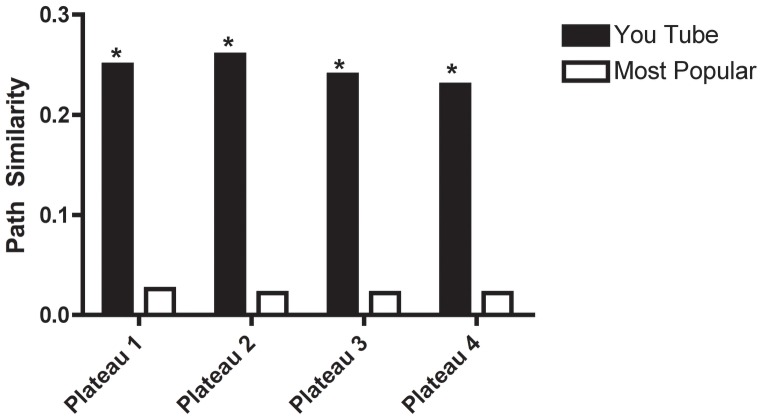
Semantic similarity of YouTube comments to established signs and symptoms of dextromethorphan use. The black bars refer to “Robo-tripping” videos from YouTube, the white bars to the most popular videos (see sec:Methods). All values for path similarity are calculated relative to the signs and symptoms mentioned in [25].

The primary goal of this study was to investigate whether signs and symptoms of drug use could be recovered from YouTube. The text presented in Table 2 strongly resembles the effects reported in [7], [25]. To determine whether this similarity is significant and specific to our corpus, we computed the path similarity between the corresponding rows in Tables 1 and 2 and compared that path similarity to words from an equivalently sized random sample of the most popular videos on YouTube. The semantic similarity between our corpus and [25] is significantly greater than the semantic similarity between a random sampling of words on YouTube and [25] (p<0.01, two-tailed Mann-Whitney test, Figure 6). This significance was assessed by comparing for each plateau the median semantic similarity between our corpus and [25] using a random sampling of words from YouTube as a control.

**Table 2:** Words specific to each plateau.

Plateau	Specific Words
100–250 (Plateau 1)	bleeding, addictive, complications
250–400 (Plateau 2)	drunkhigh [sic], buzzed, psychotropic, ache, antidepressant, debilitating, nausea, stimulating, diarrhea, hallucinogen, smashed, numb, zombiewalk
450–800 (Plateau 3)	breathing, condition, memory, nothingness, depersonalized, experimenting, slurred, recover, stoned, drunkstoned [sic], overdosed, dissociates, shakes, oded, dying, gibberish
>800 (Plateau 4)	hangovers, vision, blackout, vomiting, psychological, abdominal, dissociated, numbs

The table shows the words from YouTube comments that had a significantly high tf-idf score and were classified as semantically related to medicine according to the criteria in Table 3.

## Discussion

### Conclusions

This paper demonstrates information about commonly ingested doses of DXM and their side effects can be retrieved from YouTube comments using techniques from information retrieval and natural language processing. The benefits and reliability of utilizing natural language processing for clinical purposes have been shown in other clinical applications, for example in identifying postoperative complications in patients undergoing inpatient procedures [26].

Analyzing social media could provide data to answer questions about the recreational use of substances that would be difficult to obtain through other means. Moreover, the same techniques may apply to the analysis of other topics in social media and to other types of medical data in textual form. This approach may facilitate the automatic extraction of healthcare information from free-text, such as social media or unstructured portions of electronic health records. This poses an opportunity to supplement existing knowledge as well as potentially generate new knowledge around substance abuse.

More broadly, the analysis of anonymous comments from social media may be useful for the syndromic surveillance of other public health issues. Mental health disorders are associated with particular patterns of interactions [27] and communication [28] online. This analysis may also provide a means to describe diseases by making their current textual descriptions computable. Prior work on mining text to discover molecular markers or constellations of symptoms focussed on databases of published scientific works [29]. In the case of substance use, where users can be reluctant to admit their identities, anonymous forums can be a good source for detecting symptoms otherwise undetected.

### Limitations

A comparison of social media with more traditional sources of medical information highlights several limitations of this study. Traditional sources for data on recreational drug use include national surveys, reports from poison center calls and voluntary reports from physician encounters. Unlike national surveys, the relationship between YouTube users and the general population is not known. Unlike reports from poison center calls or physician reports, the drug effects are not assessed by trained experts. Furthermore, comments from YouTube are not verifiable, and may be hyperbolic or sarcastic.

Despite these limitations, data that are properly gathered from YouTube can counter the weaknesses of more traditional data sources. Data can be acquired from Youtube rapidly and inexpensively, and at such volume as to remove many statistical biases. Moreover, YouTube comments are more likely to acquire data about mildly intoxicated individuals who are not experiencing enough adverse effects to engage the healthcare system. Finally, the substantial agreement with published reports suggests that discussions about drugs can provide relevant, usable data.

A large limitation is that there is no way to verify that the doses of DXM mentioned in the comments are the doses that the writers of the comments ingested. Some estimates of dxm doses may be inaccurate or false. This limitation is a general problem with using social media for toxicology. In contrast, case reports from emergency rooms can have laboratory studies to verify serum levels, which much stronger evidence of ingestion of a certain dosage.

The methods used here also have limitations, which are general issues in natural language processing. Treating sentences as bags of words overlooks the structure of human language, such as how word order changes meaning. Overlooking word order obscures the context of each word and context often alters a word's meaning. Preprocessing, for example removing stopwords and punctuation, does change the distribution of words. For example, in plateau 2 of Table 2, removing punctuation created words such as “zombiewalk”. The word “zombiewalk” likely refers to the ataxic “zombie-like” gait that can occur at with DXM use. [25]. “Drunkhigh,” before pre-processing was likely “drunk-high” or “drunk high” with a space. The nature of YouTube comments further exacerbates this creation of spurious words. YouTube comments often contain spelling errors, abbreviations, and misspellings that could be intended or accidental. It is possible that some comments are hyperbolic or sarcastic. We did not control for the tone of the YouTube comment.

Some words in Table 2 are semantically related to medicine but are unlikely to be semantically related to the use of DXM. For example, “bleeding” is specific to plateau 1. Here, “specific” means that it appears more frequently in plateau 1 comments than in comments for any other plateau. The appearance of “bleeding” in plateau 1, however, does not mean that YouTube users discussing DXM at plateau 1 dosages experienced bleeding. Rather, the word “bleeding” is included because it is a keyword specific to plateau 1. That is to say, “bleeding” is statistically specific even if it is unlikely to be semantically relevant to dxm. Intricacies such as this highlight the considerable work that remains in building robust computational representations of semantics.

Finally, this study considered each YouTube comment in isolation. We did not consider the discourse semantics or pragmatics of the comment. While extended discussions are uncommon in YouTube, small threads of related comments are observed, and the context of these threads is lost using our approach.

## Methods

**Figure 7:**
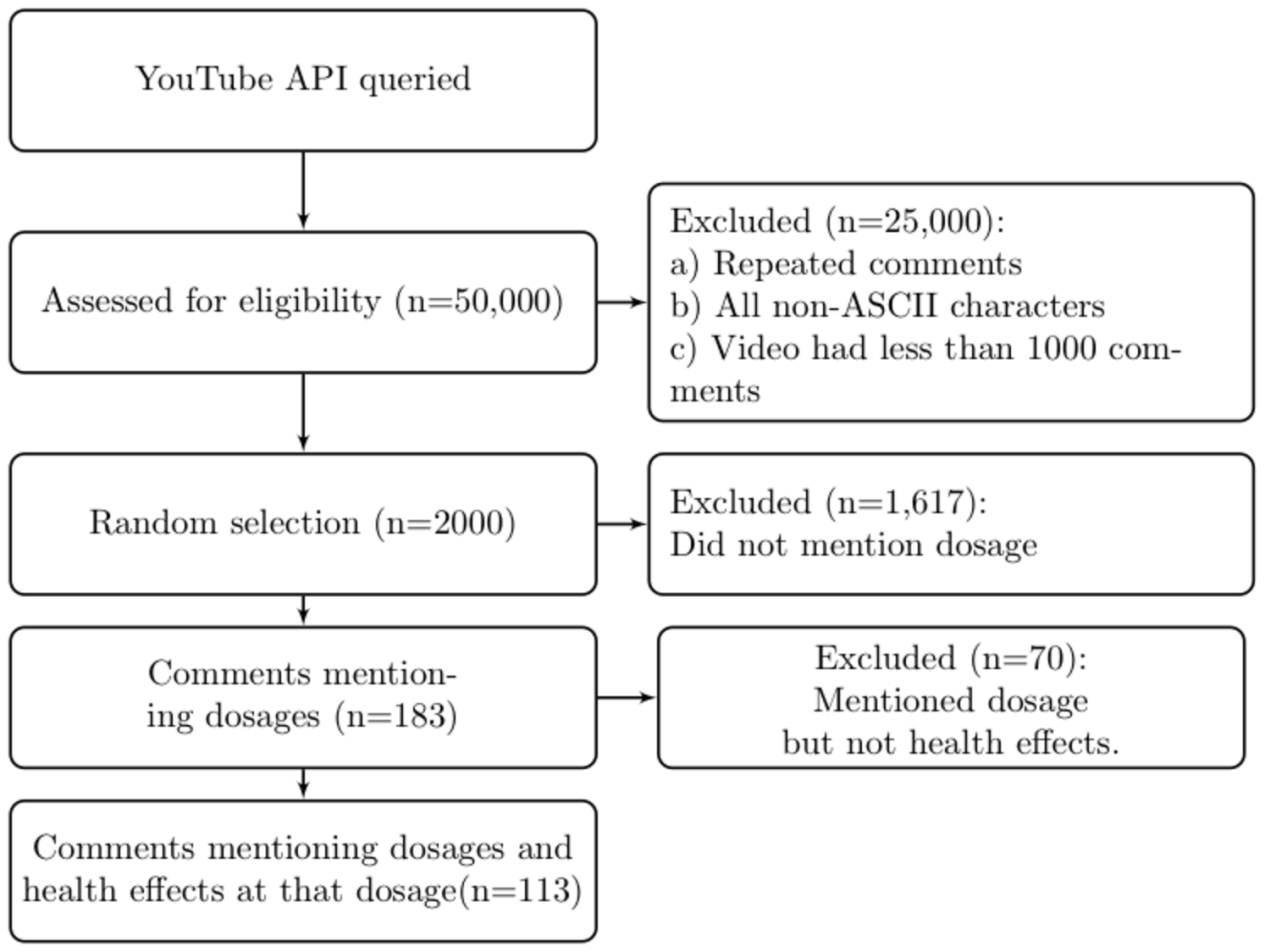
Extraction of YouTube Comments for Analysis.

Figure 7 shows the flow of analysis of YouTube comments, beginning with the extraction of comments from YouTube and concluding with the extraction of medically relevant keywords from a suitable subset of those comments.

### Corpus Development

We retrieved YouTube comments by using a Python interface (wrapper) to the YouTube API (Application Programming Interface). The API returns up to 50 videos per search and 1000 comments per video. To retrieve videos from YouTube related to use of DXM, we passed the search terms *robotrip* and DXM *trip* to the API. The results from both queries were combined and resulting database de-duplicated to yield 7500 comments. This database follows CouchDB standard and access can be granted upon request. Apache CouchDB, commonly called CouchDB, is a NoSQL (non-relational) database that stores its data in JSON format.

### Random Sampling

We randomly chose 2000 comments to analyze. The comments for analysis were a subset chosen randomly from the entire CouchDB database as follows. When each comment was added to the CouchDB database a 16-digit key from a random number generator between 0 and 1 was assigned to it. To randomly extract a certain fraction of comments, all comments with keys below the desired fraction are returned. For example to extract 10% of the database, all comments with keys between 0.1 and 0.2 could be returned. This approach was chosen because it allows one to repeatedly draw the same randomly chosen sample. This is very useful to verify random sampling as the database grows. The comments can be from any point since the inception of YouTube, but the majority of the videos were published within the past three years. All scripts used in corpus development and subsequent analysis are available at http://www.github.com/mac389/ytpy.

### Data Preprocessing

Unprocessed YouTube comments were not suitable for our analyses because they contained high amounts of noise. To make them more suitable for processing, we removed stopwords, all non-ASCII characters, and all non-alphanumeric characters. ASCII refers to the American Standard Code for Information Interchange encoding scheme, which includes definitions for 128 characters, 33 of which are non-printing control characters, for example to indicate a new line. Once the corpus was cross-indexed by plateaus, all numeric characters were also removed.

Stopwords refers to words that are removed from corpora before analysis because they are too common to identify specific bodies of text. Ubiquitous words could be removed during the analysis by excluding words whose tf-idf score is near zero. However, this risks skewing the calculation of tf-idf.

We removed two classes of ubiquitous words. First, we removed English, French, and Spanish stopwords as defined in Python's NLTK (Natural Language Toolkit). Second, we removed stopwords from a custom list (ToxTweet website. Available: https://www.dropbox.com/s/bj20vb5gjo69nmt/stopwords). The custom list included two classes of words- those formed from elisions in punctuation, such as *don't*, and canonical internet abbreviations such as *lol*, *jk*, and *ftw*. The first class ensures that variants of previously defined stopwords are also removed. The second class was removed because they contained semantic information that was irrelevant to the current study.

All characters that could not be encoded in ASCII were removed. This excludes all text that cannot be written with the Latin alphabet and so removes comments that use other scripts like Cyrillic or Devnagari. It does not exclude comments in Russian or Hindi that are transliterated into the Latin script.

### Tf-idf

The tf-idf score of a document represents the degree to which a term is more associated with one document in a group of documents than all the others. After preprocessing the data as described in the previous section, the comments were divided into 4 categories based on the dosages mentioned in the comments. These categories were chosen to correspond with the categories mentioned by [25]. The text from all comments was pooled within each category (plateau). In this scheme term frequency thus refers to how often a comment appears within a category. Inverse document frequency depends on how many categories (plateaus) mention that term.

The tf-idf of a term, 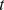, in a document, 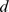, from a corpus, 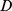, quantifies how unique that term is to that document (Equation 2).
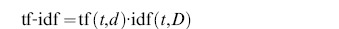


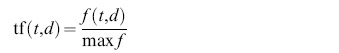


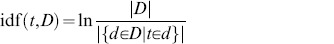
(2)


In Equation 2, the frequency is normalized by the length of each document to prevent a bias towards longer documents. The denominator of the third line denotes the subset of all documents that have the indicated term, 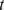. Despite the similarity in formalism to entropy, tf-idf is only a measure of term specificity. We use it here to identify the most characteristic words associated with each plateau. We do not equate characteristic with *informative*.

### Keyword Extraction

To identify words that were significantly associated with each plateau, those words whose tf-idf score was above an empirically determined threshold were extracted. For each plateau, the threshold (see previous subsection Tf-idf ) was set as the score at the 75th percentile of the empirical cumulative distribution function of tf-idf scores for that plateau. To identify words that were significantly associated with each plateau and semantically related to drug use, the keywords were manually subdivided into three categories- Yes, No, and Maybe. The category headings denote whether the words therein are semantically related to drug use (see Table 3).

**Table 3:** Categories for stratifying words based on semantic relation to drug use.

Category	Definition
Yes	Likely semantic relation to medicine, such as “dizzy”
Maybe	Possible semantic relation to medicine, such as “heavy” which could refer to a somatic sensation or the weight of an object
No	Very unlikely to relate to medicine, such as “penguin” or “satan”

### Word frequency by plateau

**Figure 8:**
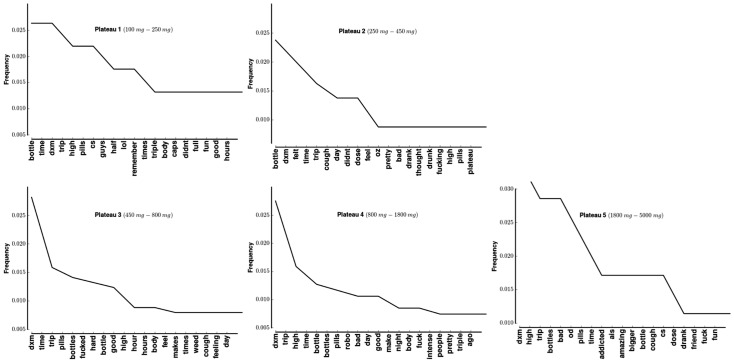
Most frequent words in YouTube comments stratified by plateau.

To investigate whether specific words from YouTube comments were associated with each plateau, the comments were divided into categories based on dosage they discussed. Figure 8 shows the 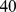 most frequent words for each of those categories.

The most frequent or second most frequent word in each panel of Figure 8 is DXM. Three words are common to the top five comments across each plateau: “DXM”, “time”, and “trip”.

**Figure 9:**
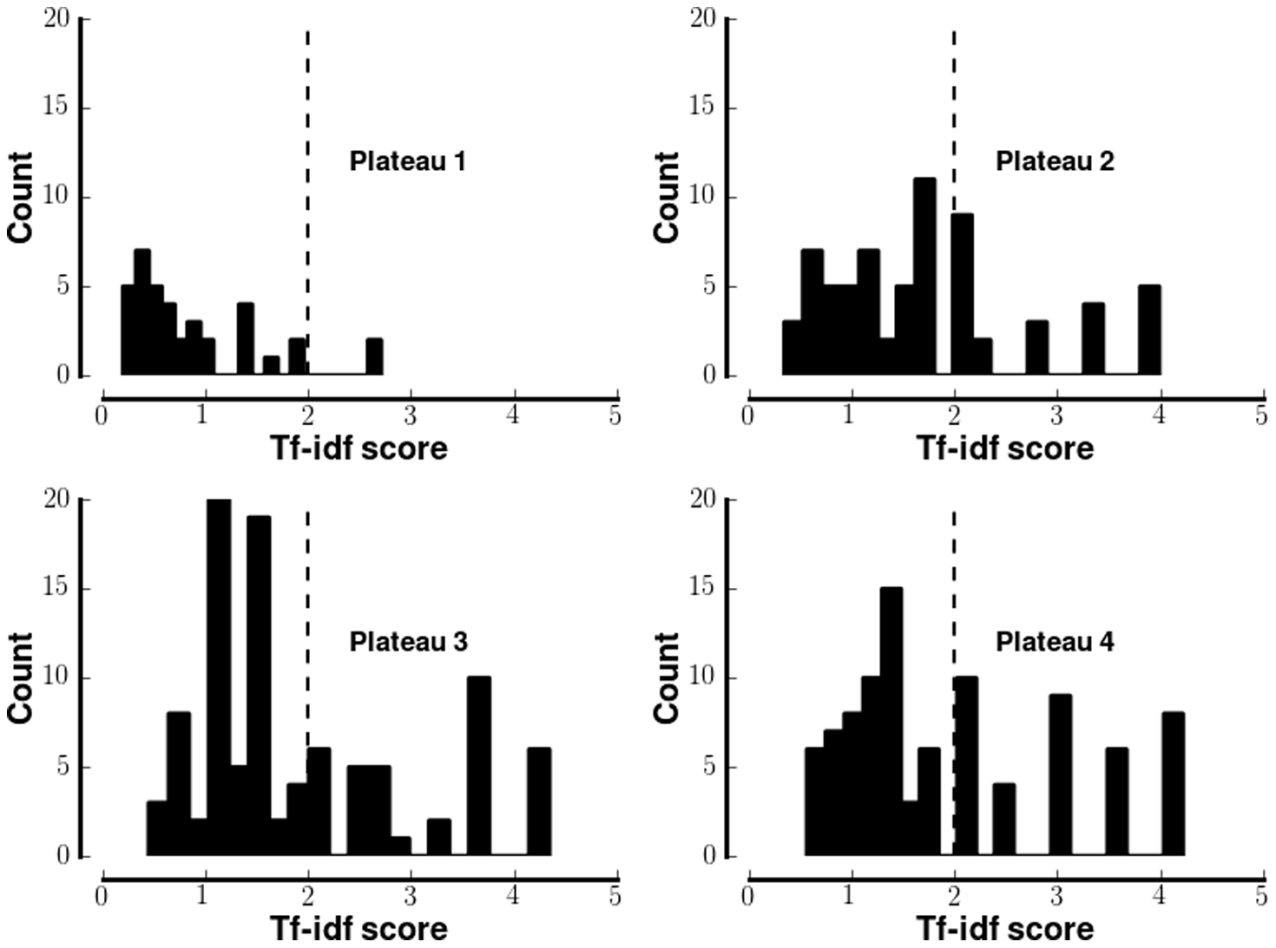
Distribution of tf-idf scores for YouTube comments. Each panel shows the distribution of tf-idf scores for YouTube comments stratified by the plateaus defined in [25]. The dotted line shows the threshold beyond which the tf-idf score indicates that the word indicates a specific plateau.

To identify systematic differences the tf-idf score for each word in the corpus was calculated (Figure 9). In calculating that score, each plateau was treated as a document.

To identify sufficiently discriminative words, a threshold tf-idf value at the 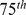 percentile of the empirical cumulative distribution function was established. This procedure generates a threshold for each plateau. For simplicity, the greatest of these thresholds was used (dotted line in Figure 9). Table 2 lists the subset of those words that were semantically related to drug use.

Table 2 shows words that are specific to each plateau and are semantically related to medicine. Most of the words correspond with the descriptions of the states found in Table 1.

### Semantic Similarity

The tf-idf identifies keywords that help distinguish one document from another. To find the subset of keywords semantically related to medicine, we then excluded words whose synonym ring (*synset*) included no medical words. The synset of a word is the collection of all words that are synonymous to it. To quantify how semantically similar to words are, we used a different distance measure: path similarity. The path similarity is a function in NLTK that returns a score that quantifies how similar the sense (semantic content) of two words are base on their path in the hyponym-hypernym hierarchy of WordNet. A hyponym is a word that is semantically related to another word but refers to more specific things. For example *ambulance* is a hyponym of *automobile*. A hypernym is a word that is semantically related to another word but refers to more general things. For example, *motor vehicle* is a hypernym of *automobile*. The score is normalized with the maximum similarity being unity.
